# WORKFORCE CHALLENGES DURING COVID-19

**DOI:** 10.1093/geroni/igad104.0693

**Published:** 2023-12-21

## Abstract

As of October, 2022 nearly 1.1 million individuals in the United States died of COVID-19 with approximately 790,000 being individuals ages 65 and older. This age group accounts for 16% of the total US population but 75% of all COVID deaths to date. Covid-19 has taxed the United States public health system resulting in clinician burnout and a lack of trust in the public health system. The demands of the pandemic physically, psychologically, and logistically overwhelmed many clinicians resulting insomnia, anxiety, depression, and increased risk for substance use and misuse. Burnout at the institutional level is associated with increased medical errors, lower quality of care, and hospital-acquired infections. Compounding the issue of burnout was the lack of public trust that subsequently developed. Facts about COVID-19 competed with misinformation of the disease, false medical cures, contradictory health messages, an overwhelmed health care system in many areas, and conspiracy theories. One solution to these concerns is to increase the use of Community Health Workers (CHC). CHCs are trusted members of the communities they serve and play an essential role in assisting in care, providing factual information, and dispelling misinformation related to health topics such as COVID-19 infection, symptoms, and vaccination. Augmenting this workforce may help the system prepare for future emergencies. This presentation will discuss the recommendations of the Federal Advisory Committee on Interdisciplinary, Community-Based Linkages’ latest report “Building Trust, Addressing Burning, and Expanding the Direct Care Workforce”.

